# Adherence to treatment for polycystic ovarian syndrome: A systematic review

**DOI:** 10.1371/journal.pone.0228586

**Published:** 2020-02-13

**Authors:** Madison Parker, Anna Warren, Sonam Nair, Marie Barnard

**Affiliations:** 1 School of Pharmacy, University of Mississippi, University, MS, United States of America; 2 Department of Pharmacy Administration, University of Mississippi, University, MS, United States of America; Zhejiang University School of Medicine Women's Hospital, CHINA

## Abstract

**Background:**

Polycystic ovarian syndrome (PCOS) is one of the most prevalent endocrine disorders of women of reproductive age. Treatment plans for this chronic condition frequently include long-term use of a combination of medication and lifestyle interventions. However, treatment outcomes are dependent on adherence to treatment regimens. This study aimed to systematically review the literature for reported adherence to treatments for PCOS.

**Methods:**

A systematic search of Embase, Cochrane, PubMed, CINAHL, PsychINFO, SCOPUS, and International Pharmaceutical Abstracts from inception until January 2019 utilizing the terms PCOS, adherence, and patient compliance was conducted. A total of 179 possible articles were identified.

**Results:**

Fourteen articles reporting adherence data were included in the review. Self-report was the most commonly reported method of measuring adherence. Adherence to lifestyle interventions, such as prescribed diets and physical activity, was reported in ten studies and adherence to medications was reported in seven studies, with some reporting both.

**Conclusions:**

Minimal data are available regarding factors associated with adherence in patients with PCOS. Diverse methods of adherence assessment are utilized. Future studies of PCOS treatments should effectively assess and report adherence data as it is essential to evaluating the effectiveness of PCOS treatments and is critically needed to guide clinician efforts to facilitate optimal outcomes for patients.

## Introduction

Polycystic ovary syndrome (PCOS) is the most common endocrine disorder of reproductive-aged females, impacting 6 to 10% of women.[1] Much attention has been given to the need for careful clinical assessment for diagnosis to develop an optimal treatment approach, with treatment modalities including medication and lifestyle management.[2] Treatment goals address a variety of symptoms from reproductive function to hirsutism and acne, as well as commonly associated issues such as insulin resistance. In addition to symptom management, treatment goals include the prevention of long-term complications associated with PCOS, such as diabetes and cardiovascular disease.[3] Treatment protocols for PCOS are complex, and include more than 160 recommendations and practice guidelines.[4] First line pharmacological treatment for menstrual irregularity and hyperandrogenism is oral contraceptive pills, with metformin recommended for management of metabolic features.[4]

Treatments for PCOS usually require a multi-component plan that require substantial patient engagement. While the literature is replete with studies examining the outcomes associated with various treatments for PCOS, it is critical to understand the role of patient adherence to the treatments to evaluate the value of the treatments for patients in the real world. This is especially important given that the World Health Organization (WHO) reports that in developed nations only 50% of patients with chronic diseases adhere to treatment recommendations.[5]

Treatment regimens are of no use if a patient does not take the medication or engage in the recommended behavior (e.g., physical activity). Treatment plans for PCOS often include medications that are known to have side effects that may impact adherence. Many PCOS patients are also asked to include lifestyle management interventions in the treatment regimen. Although noting that adherence to diet and physical activity treatment recommendations can be challenging for patients, recent summaries of the evidence for the assessment and management of PCOS reported no data related to treatment adherence.[4,6] Prior reviews have indicated that adherence to diet and physical activity treatment recommendations can be challenging for patients and is critical to achieve treatment goals.[7]

It is also important to note that adherence is a critical component of evaluating a reported treatment effect. Clearly defining and reporting adherence to treatments is essential for valid quantitative assessment of the outcome of treatments and their ability to explain clinical and economic events.[8] Inclusion of measures of treatment compliance as primary or secondary outcomes are important to support evidence-based medicine.[9] Reporting how adherent participants were to an intervention in conjunction with reporting the treatment outcome facilitates the ability to more accurately predict treatment effect and provides evidence to support patient education on the impact of adherence.

Given the multifactorial treatment recommendations for PCOS, a thorough understanding of adherence to treatment and its impact on treatment-related outcomes is needed. This project seeks to address the question of how and in what study contexts adherence to PCOS treatments is reported.

## Methods

The Preferred Reporting Items for Systematic Reviews and Meta-Analyses (PRISMA) was utilized to guide the systematic review of the literature.[10] An evidence-based list of items recommended for report in systematic reviews, PRISMA improves systematic reviews and provided the basis for the review protocol used in the current study (S1 Fig). The search strategy to identify potential articles included searches of Embase, Cochrane, PubMed, CINAHL, PsychINFO, SCOPUS, and International Pharmaceutical Abstracts. In order to identify all the available literature, no date limits were applied. The databases were searched in January 2019. The search strategy for one of these database is presented (S1 Table). A total of 179 non-duplicate articles were identified through these searches.

**Fig 1:**
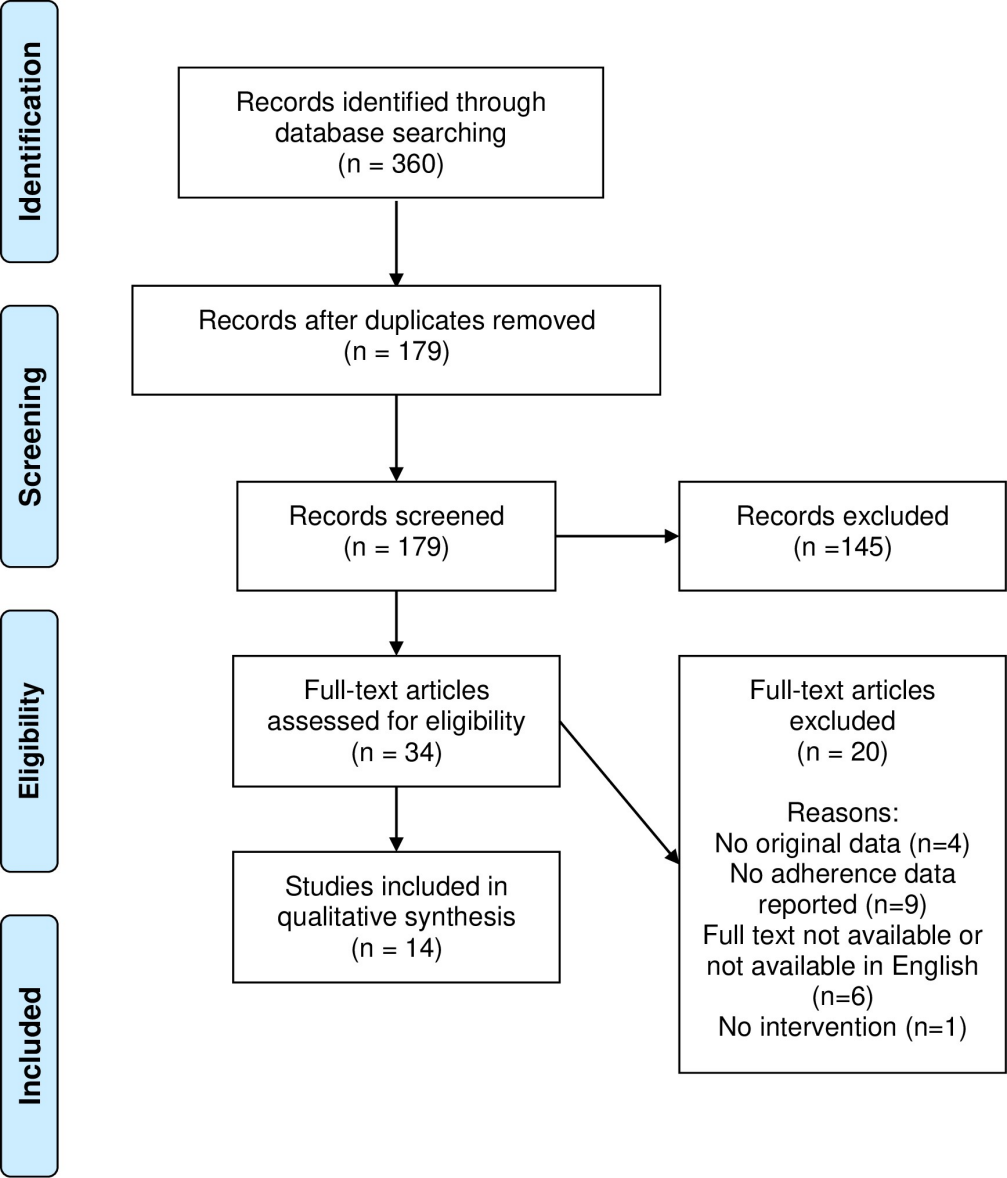
PRISMA flow diagram.

All identified articles were screened for full-text review. Inclusion criteria for full-text review were (1) focused on PCOS and (2) reported adherence or compliance to a therapy or treatment regimen. Adherence had to be reported as adherence to the intervention or treatment, not to treatment guidelines. Change in biomarkers without a measure of adherence to the treatment was not considered sufficient as it is not possible to know if the change was specifically related to utilization of the treatment or to other factors. Three authors (MP, AW, and SN) screened titles and abstracts for inclusion in the full-text review, with a fourth author (MB) resolving any discrepancies. A total of 145 articles that did not focus on a PCOS treatment nor address adherence were excluded. The remaining 34 articles were identified for full-text review. The data charting process was conducted via a Qualtrics data entry form. The form was tested by the team, with all team members completing reviews of several articles to ensure the abstraction process was uniform. Data items abstracted included study design and duration, population (inclusion criteria, sample size, demographics, setting), intervention and comparison treatment descriptions, treatment allocation, blinding, description of adherence data and how adherence was measured, results related to adhere and other study outcomes by treatment group, including time points for which outcomes are reported (length of follow-up), how missing data was handled, management of missing participants, key conclusions, and funding source. The charting was conducted by three authors (MP, AW, and SN) and a fourth author (MB) resolved any discrepancies. Additionally, references in all articles for which a full-text review was completed were scanned for potential identification of any references that may not have been identified in the original search. No additional references were identified. After full-text review, 20 articles were excluded. Studies were excluded after full text review if they did not have original data, did not report patient adherence data, did not report on an intervention, or did not have the full text available in English. Adherence was the main outcome abstracted. The PRISMA flow diagram describes the article selection process (Fig 1). A narrative summary of the evidence was then developed utilizing an inductive content approach.[11] Review of the abstracted data was conducted by three authors separately (AW, MB and SN) and then reviewed and confirmed by all authors. Study quality, including risk of bias at the study level, was evaluated using the PEDro scale items.[12] This scale was employed as it is utilized with many physiotherapy reviews and has been validated for use in evaluating the methodological quality of pharmacological trials as well.[13] Two authors (MB and SN) independently scored the studies, and disagreements were resolved through a discussion with the two other authors (MP and AW).

## Results

### Study selection, quality and participants

The 14 articles selected for review are summarized in Table 1. The publications included information about 835 females with PCOS. Eleven of the studies were randomized controlled trials. The other three included a quasi-experimental study, a retrospective cohort study,and a cross-sectional survey study. Study quality was assessed utilizing the PEDro scale which assesses factors primarily associated with the study design and focuses on the studies’ main outcomes, which were not necessarily adherence to the intervention. Study quality varied across the articles, with blinded providers and assessors clearly being the factors least likely to be satisfied (Table 2). Findings are summarized in four areas: limitations of reported adherence data, adherence to lifestyle interventions, adherence to medication interventions, and outcomes assessed concomitantly with adherence.

**Table 1:** Summary of articles included in the review.

Author (year)	Research Design	Sample and Sample Size	Intervention/ Duration	Adherence Measure Description	Adherence Outcomes	Study Outcomes
Allen et al., 2005[14]	RCT	• n = 36 • Inclusion criteria: Hyper-androgenemia, no evidence of androgen secreting tumor, oligomenorrhea, and obesity	• Group 1 (n = 15): oral contraceptive pills (OCPs) • Group 2 (n = 16): metformin • Follow-up: 6 months	• Medications: Patients were considered non-compliant if they did not bring their remaining medications to a visit for a pill count or if the pill count revealed more than 25% of pills prescribed for the 3-month interval remained	• OCP group had higher compliance (87% compliant) compared to metformin group (69% compliant)	• Metformin was as effective as OCPs in regulating menses, improving hirsutism and acne, and reducing androgen levels over 6 months. • In participants who achieved weight loss: OCPs were comparable to metformin in improvement of some markers of long-term metabolic risk
Arentz et al. 2017[15]	RCT	• n = 122 • Inclusion criteria: reproductive age, PCOS diagnosis, BMI, not on certain medications	• Group 1 (n = 62): lifestyle intervention • Group 2 (n = 60): lifestyle intervention and herbal medicine • Follow-up: 3 months	• Exercise: Self-reported intensity of exercise (mild, moderate, vigorous) and number of minutes per week. Reported biweekly and at an interview at week 12 • Diet: Average number of self-reported servings of vegetables and fruit per day and number of high energy, nutrient sparse meals per week • Herbal medicine: Tablet count with return of herbal bottles at 3 months	• Exercise: 89 of 122 (73%) self-reported at least 150 min exercise per week • No significant differences between groups for number exercising 150 min or more per week • Diet: 105 of 122 (86%) reported health conscious dietary decisions. • No significant differences between groups for number reporting health conscious dietary choices • Herbal medicine: not reported	• The proportion of participants with normal menstrual cycle length (20–34 days) in the herbal medicine plus lifestyle group was significantly greater compared with controls • Fasting insulin was significantly lower for participants taking herbal medicine in addition to lifestyle compared with controls • Participants taking the herbal medicine tablets plus lifestyle recorded a significantly greater reduction in depression, anxiety and stress scores compared with those in the lifestyle intervention only arm
Atiomo et al. 2009[16]	RCT	• n = 11 • Inclusion criteria: Age 35+, obese, infrequent or absent menstrual periods, hirsutism, infertility, and PCOS diagnosis	• Group 1 (n = 6): low 600 calorie deficit glycemic index diet • Group 2 (n = 5): 600 calorie deficit healthy eating diet • Follow-up: 6 months	• Diet: Documented attendance at monthly diet clinic meetings and a food diary	• Data not reported by treatment group • 4 of 8 women attended all 6 required diet clinics • 8 of 8 women completed food diaries but no compliance to the diet is reported • 3 patients dropped out of study and no data is provided for these patients	• Participants randomized to low glycemic index diet had a greater decrease in endometrial thickness and greater increase in the number of menstrual cycles
Cooney et al. 2018[17]	RCT	• n = 33 • Inclusion criteria: PCOS, BMI = 27–50, depressed	• Group 1: Weekly 30-minute sessions of cognitive behavioral therapy (CBT) and lifestyle modification program of in-person individual weekly nutrition and exercise counseling for 16 visits • Group 2: Same lifestyle modification program without CBT • Follow-up: 16 weeks	• Diet and Exercise: Daily food intake and exercise logs reviewed at each counseling session • Program: Lost to follow-up—withdrew, unable to commit, or unable to complete intervention	• Lost to follow-up: 8 in CBT and lifestyle arm and 4 in lifestyle only arm • Session attendance: both groups attended an average of 14 of the 16 sessions • Reported exercising in the past week: 84.5% CBT and lifestyle vs 74.6% lifestyle only • Reported meeting weekly exercise goal: 59% CBT and lifestyle vs 38% lifestyle only • Median number of minutes of exercise per week: 102 minutes CBT and lifestyle vs 90 minutes lifestyle only • CBT and lifestyle participants brought food diary to 83% of weekly visits vs lifestyle only participants brought food diary to 66% of weekly visits	• Participants in the CBT+ LM category were more likely to meet their exercise goals & keep food diaries • The CBT+LS group showed more than twice as much weight loss per week & better scores on the psychological assessments
Foroozanfard et al. 2017[18]	RCT	• n = 60 • Inclusion criteria: Overweight or obese; aged 18–40; PCOS • Stratified by BMI and age and then randomized	• Group 1 (n = 30): low calorie DASH (dietary approaches to stop hypertension) diet • Group 2 (n = 30): control diet • Follow-up: 12 weeks	• Diet: Dietary recalls collected at baseline, and weeks 3,6,9, and 12 during the intervention. • Exercise: Records collected at baseline and weeks 3,6,9, and 12 during the intervention	• Nutrient level differences between the groups are described but no adherence data for diet nor for physical activity	• DASH diet participants significantly decreased BMI, AMH, insulin, HOMA-IR, HOMA-B, serum SHBG, FAI and plasma MDA, and significantly increased QUICKI and plasma NO levels compared with the participants following a low-calorie control diet; however, the DASH diet did not affect FPG and other hormonal profiles
Hoeger et al. 2004[19]	RCT	• n = 38 • Inclusion criteria: diagnosed PCOS; obese or overweight; normal TSH, prolactin, FSH, and metabolic profile	• Group 1: metformin 850 mg two times per day • Group 2: lifestyle modification program with placebo two times per day • Group 3: lifestyle modification and metformin two times per day • Group 4: placebo two times per day	• Medication: Monthly capsule count • Program: Study dropout was identified as a compliance measure	• Capsule count data not reported • Overall dropout was reported as 39% • 20% of the lifestyle modification arms dropped out	• Modest weight reduction was found in all treatment groups, with the most significant reduction occurring with the combination of metformin and lifestyle intervention. Significant androgen reduction occurred in the combination group only. Ovulation rates did not differ significantly between groups. However, when data were analyzed by presence or absence of weight reduction in subjects, independent of treatment group, the estimated odds ratio for weight loss was 9.0 (95% confidence interval 1.2–64.7) with respect to regular ovulation.; 39% of subjects dropped out. 15% because of adverse reactions, 16% because of time commitment
Karamali et al. 2018[20]	RCT	• n = 60 • Inclusion criteria: PCOS; taking metformin tablet at the initial dose of 500 mg, which was increased in a stepwise manner during the first 3 weeks to a total of 1500 mg day for all subjects; participants were matched on BMI, age and phenotype	• Group 1 (n = 30): prescribed diet with 35% protein from animals and 35% protein from textured soy • Group 2 (n = 30): prescribed diet with 70% protein from animals • Follow-up: 8 weeks	• Diet: 3-day food records at baseline, 2 weeks, 5 weeks and end of trial; diet compliance also monitored once a week via telephone interviews	• Neither food record data nor compliance as captured by weekly interviews is reported • Analysis of the food records is reported and indicates no difference in energy, carbohydrates, protein, and fats but there were significant differences in dietary intakes of saturated fatty acids, polyunsaturated fatty acids, and dietary fiber	• All 60 participants completed the trial • Reported that adherence to the test diet, compared to the control diet, resulted in significant decreases in serum insulin levels • Reported that adherence to the test diet, compared with the control diet, resulted in significant decreases in BMI, fasting glucose, testosterone, insulin, insulin resistance, etc.
Karjane et al. 2012[21]	Retrospective cohort study	• n = 173 • Inclusion criteria: Available charts and well-documented PCOS diagnosis	• Group 1 (n = 109): metformin • Group 2 (n = 63): oral contraceptive pills (OCP) • Follow-up: 12 months	• Medications: Persistence to treatment determined by patient report of taking the prescribed medication	• Metformin group persistence: 3 months 57.8%, 6 months 43.9%, 12 months 31.2% • OCP group persistence: 3 months 57.1%, 6 months 38.1%, 12 months 21.7%	• Patients with hirsutism were more likely to be persistent to metformin • Factors associated with persistence with OCPs included diabetes, younger age, and a lower BMI
Ladson et al. 2011[22]	RCT	• n = 114 • PCOS diagnosis, aged 21–39, in good health, currently off confounding medications • Randomized in a 1:1 allocation ratio in permuted blocks and stratified by center and prior metformin exposure status	• Group 1 (n = 55): Metformin and lifestyle (caloric restriction and exercise) intervention • Group 2 (n = 59): Placebo and lifestyle (caloric restriction and exercise) intervention • Follow-up: 6 months	• Program: Lost to follow-up—withdrew, unable to commit, or unable to complete intervention • Exercise: Polar heart rate monitor that recorded heart rate, duration, and caloric expenditure was worn; daily activity log once per month	• Lost to follow-up: 33 in metformin and lifestyle arm and 43 in placebo and lifestyle arm • Number of workout sessions per week average 2.5+-1.2 in the metformin group and 2.5 +-1.8 in the placebo group	• No difference in ovulation rates, exercise parameters, diastolic blood pressure, hirsutism or acne scores, overall well-being scores, ovarian volume of size of the largest follicle • Metformin arm had significantly greater increase in bone mineral density, central-to-total body fat ratio and greater decrease in insulinogenic index whereas lifestyle only arm had significantly greater AUC glucose
Li et al 2011[23]	Cross-sectional	• n = 90 • PCOS treated with medications, absence of other endocrine disorders, independent cognitive ability with no history of physical or mental illness	• No intervention	• Morisky-Green test administered as an interview that resulted in an indicator for ‘good adherence’ to medication and doctor’s advice	• 23 out of 90 (25.55%) were adherent	• Many PCOS patients exhibited non-compliance which was associated with patient's convenience of medical treatment, BMI and concerns about adverse drug reactions.
Liao et al. 2008[24]	Observational	• n = 35 • PCOS and BMI >25, under 40 years, non-smoking, could not be taking any medication, or have cardiovascular disease, hypertension, thyroid disease, diabetes, or any endocrinopathy	• Subjects followed a program of regular exercise comprised of brisk walking at least 3x per week at a self-selected brisk pace for at least 20 minutes • Follow-up: 6 months	• Exercise: Monthly verbal report; exercise record charts—entry for each episode and its duration returned monthly; assessment of oxygen consumption to provide an objective measure of compliance by providing an individualized test of whether expected exercise-induced physiological adaptation occurs	• 12 participants completed the brisk walking program and carried out 80% of their target exercise volume on average • 11 participants did not begin or did not complete the program and this group completed 6% of the target exercise volume on average • 12 participants did not present for re-assessment and no exercise volume data is available • Complete agreement between self-report of exercise participation and physiological data	• Significant reduction in waist-to-hip ratio in the completers; • Non-completers had significantly lower BDDE-SR scores than either completers or non-exercisers. • Pre and post data suggest that a self-directed brisk walking program can reduce body image distress in women with PCOS
Otta et al. 2010[25]	RCT	• n = 30 • PCOS, no meds 3 months prior to study, excluded any other possible cause of hyper-androgenism	• Group 1 (n = 15): metformin • Group 2 (n = 15): placebo • All told to exercise, given a nutritional plan of 1500 calories • Follow-up: 4 months follow-up	• Program: Monthly visits to evaluate clinical, anthropometric parameters, treatment compliance and adverse events	• One participant in metformin group withdrawn for lack of adherence to treatment • No adherence to diet or exercise reported.	• Statistically significant reduction in total testosterone in metformin group
Turner-McGrievy et al. 2014[26]	RCT	• n = 18 • PCOS, obese/ overweight, 18–35, trying to conceive for at least 6 months, not currently taking fertility-enhancing medications except metformin	• Group 1 (n = 9): vegan diet • Group 2 (n = 9): low calorie diet • Both groups received 2 counseling sessions with RD, then weekly emails with diet lesson and tailored message about their weight loss and reported dietary adherence. Each group also had a private Facebook (FB)group to interact with one another and read messages from study dieticians • Follow-up: 6 months	• Diet: Weekly questionnaire (24 total) assessing dietary adherence and submission of the questionnaire was considered duration of exposure to the intervention • Program: Self-report of accessing study materials (email, FB, newsletters)	• Adherence reported as energy and macronutrient intake • 67% of all participants reported at 3 months that they accessed the study materials sometimes or very often • Vegan participants engaged FB a median of 4 times per person and low-calorie participants engaged a median of 6 times per person • Vegan group reported reading more of the weekly electronic newsletters median 19 of 24 newsletters than the low-calorie group 8 of the 24 newsletters	• Participants in the vegan arm lost more weight at 3 months compared to those in the low-calorie group but this difference was not observed at the 6-month follow-up
Vizza et al. 2016[27]	RCT	• n = 15 • PCOS, age 18–42, not currently doing resistance training, not pregnant/bf, no CVD, kidney, respiratory, HT or cancer, no cigarette use for 6 months, no acute or chronic conditions that would rule out exercise, English speaking	• Group 1 (n = 8): 2 supervised training sessions/week and 2 home-based exercise sessions/week for 12 weeks • Group 2 (n = 7): Usual care, followed their usual physical activity • Follow-up: 13 weeks	• Exercise: Log-books -computed as the number of sessions attended divided by the number of sessions offered	• Adherence to training in experimental group was 76% for supervised training and 43% for home-based training, 60% overall	• The PRT group reported a significant increase in body weight and BMI compared to the control group. There was also a significant reduction in waist circumference (p = 0.03) and a significant increase in lean mass (p = 0.01) and fat-free mass (p = 0.005), indicating that the weight gain was due to muscle hypertrophy, PRT group reported a significant reduction in HbA1c over time compared to the control group.

**Table 2:** Quality of the studies included in the systematic review.

	**PEDro Scale Criteria**											
**Study**	**1**	**2**	**3**	**4**	**5**	**6**	**7**	**8**	**9**	**10**	**11**	**Total Score**
Allen et al. 2005	+	+	+	+	?	?	?	+	?	+	+	7
Arentz et al 2017	+	+	+	+	-	-	+	+	+	+	+	9
Atiomo et al 2009	+	+	-	?	-	-	+	-	-	+	-	4
Cooney et al. 2018	+	+	-	+	-	-	-	-	-	+	+	5
Foroozanfard et al. 2017	+	+	+	+	+	-	+	+	+	+	+	10
Hoeger et al. 2004	+	+	+	+	+	+	-	-	-	+	+	8
Karamali et al. 2018	+	+	+	+	+	+	?	+	N/A	+	+	9
Karjane et al. 2012	+	-	N/A	N/A	N/A	N/A	N/A	N/A	N/A	+	+	3
Ladson et al. 2011	+	+	+	+	+	+	?	-	+	+	+	9
Li et al. 2011	+	-	-	-	-	-	-	+	N/A	+	-	3
Liao et al. 2008	+	-	-	N/A	-	-	-	-	-	+	+	3
Otta et al. 2010	+	+	+	+	+	+	?	+	?	+	+	9
Turner-McGrievy et al. 2014	+	+	+	+	+	-	?	-	+	+	+	8
Vizza et al. 2016	+	+	+	+	-	-	-	-	+	+	+	7

Column numbers correspond to the following PEDro scale criteria

1. Eligibility criteria specified

2. Subjects randomly allocated to groups

3. Allocation was concealed

4. Groups similar at baseline

5. Blinded subjects

6. Blinded treatment providers

7. Blinded assessors

8. Measure of key outcome obtained from at least 85% of subjects initially allocated to groups

9. Intent-to-treat analyses utilized

10. Between group comparisons conducted

11. Point measures and measures of variability presented

+ Indicates criterion was clearly satisfied;—indicates criterion was not clearly satisfied

? indicates that it is not clear if criterion was satisfied

N/A indicates not applicable

### Limitations of reported adherence data

The most common reason for studies to be excluded at the full text review stage was lack of reported adherence data. While many of the studies indicated that adherence to the interventions was assessed, the data was not reported. All studies that reported adherence in any way were included. Logs of physical activity and/or diet,[16–18,20,22,24,27] self-reported recall,[15,18,20,21,23,24,26,28] and pill counts[14,15,19] were the most common methods to measure adherence. Twelve out of the 14 studies utilized self-reported data to measure adherence. Some of the studies did collect biological measures that could examine the veracity of the self-reported adherence, however only two reported analysis of this kind.[20,24] Although multiple studies collected data related to adherence to a physical activity intervention, only one utilized a physical activity tracking device to verify the data.[22]

### Adherence to lifestyle interventions

A variety of lifestyle interventions were included as treatment arms. Nine studies included a dietary intervention.[15–20,22,25,26] Dietary interventions studied included low calorie diets, a vegan diet, the DASH diet, and a soy-based diet. Adherence rates ranged from 86% reporting they made health-conscious dietary decisions[15] to 50% attending all of the required diet clinic meetings,[16] to 67% reporting they had accessed the dietary guidance materials.[26]

Seven studies reported physical activity interventions.[15,17,19,22,24,25,27] Adherence to physical activity ranged widely depending on the type of intervention. For example, Vizza et al. reported that adherence to supervised training was 76% compared with 43% for independent home-based exercise.[27] Arentz et al. found that 73% of participants self-reported at least 150 minutes of exercise per week.[15] Cooney et al. found that 59% of participants who participated in cognitive behavioral therapy in addition to lifestyle counseling met exercise goals compared to 38% in the lifestyle counseling only group.[17] Ladson et al. reported the average number of workout sessions per week by treatment group and data from an activity monitor.[22] Liao et al. reported that 34.3% of participants completed the walking program and of those, 80% carried out 80% of their targeted exercise goal.[24]

### Adherence to medication interventions

Five of the studies included in this review utilized a medication as at least one of the interventions.[14,19,21,22,25] Two medications were studied, metformin and oral contraceptives (OCP). Three of the medication intervention studies utilized a placebo control group.[19,22,25] Allen et al. reported that over a six month follow-up period the OCP group had higher compliance (87%) compared to the metformin group (69%).[14] Karjane et al. reported that persistence in the metformin group went from 57.7% at 3 months to 31.2% at 12 months, compared to persistence in the OCP group which went from 57.1% at 3 months to 21.7% at 12 months.[21] Although the Li et al. study did not implement an intervention, this cross-sectional study found that only 25.55% of the participants were adherent as assessed by the Morisky-Green test.[23] Three studies that included a medication treatment arm did not report data about adherence to the medication.[19,22,25] Instead, they reported study drop-out rates and it is unclear that drop-outs were non-adherent.

### Concomitantly assessed outcomes

Adherence is a process measure, a step between the intervention and the desired health outcome. In the studies that reported adherence data, a variety of outcomes were examined. The most common outcome assessed was the treatment impact on hirsutism which was assessed in eight of the studies. Other commonly targeted outcomes included obesity or BMI (nine studies), insulin resistance (six studies), testosterone levels (five studies), and hyperandrogenism (four studies). Five studies examined the intervention impact on ovulation and four reported infertility outcomes. Outcomes were not limited to biological assessments. Three studies included psychological outcomes, including quality of life and depression, assessed via questionnaires.

## Discussion

PCOS is a complex condition with multiple treatment options, including medications and lifestyle interventions. Although adherence to these medications and diet and exercise interventions is critical, few studies investigating these treatments report adherence data. For those studies that do report adherence data, there is little consistency as to how adherence is measured and reported.

This review suggests that there are wide variations in adherence to treatments for PCOS. Adherence rates ranged from 21.7%[21] to 86%.[15] This wide variation is likely driven by multiple factors. Studies differed in the length of follow-up, treatment type, and adherence measurement method. These differences likely contribute to the variation in reported adherence rates, making it difficult to understand how effective any given treatment may be or how adherence any given patient may be to a particular treatment. Patients face many barriers to successfully adhering to a recommended treatment regimen. Barriers to adherence include financial barriers, medical access challenges, perception and knowledge of the disease, and insufficient patient education by the healthcare provider team.[28] Clinicians should consider these factors when planning treatments and when assessing treatment effect. It is of value to examine the outcome data in studies that reported adherence data because the true treatment effect can be more accurately estimated. In the absence of adherence data, reported treatment effects may be underestimating the potential clinical impact of a treatment. A recent review suggests that at least two methods of measuring adherence should be included in every study as there is no “gold standard” method for measuring adherence.[29] Future studies of PCOS treatments should measure and report adherence to each treatment component to facilitate a better understanding of the role adherence may play in treatment effectiveness. Clinicians should consider whether adherence is reported when evaluating the reported outcomes of a particular treatment.

Systematic reviews offer several strengths, including a comprehensive search of the literature, with the result being a synthesis of the most available information. Additionally, the broad scope does not include only a single study design but includes multiple study types.[30] While this review offers insight into adherence to treatments for PCOS, it is critical to recognize that the studies included in this review had several limitations. The relatively low scores on the PEDro Scale assessment indicates that there are limitations to the study designs for many of the reviewed studies. There are also limitations to this systematic review itself. As with all systematic reviews, this review is impacted by publication bias. While we did review abstracts, it is possible that our search strategy did not identify all studies that should have been included. It is possible that studies reported adherence but the search failed to locate these studies. We further note that we only included literature that was available in English which may result in missing studies that address some of the limitations we identified in the literature we reviewed.

Reported adherence to PCOS treatments varies substantially. Futures studies should include and report at least one measure of adherence to every treatment evaluated. Clinicians should be aware that treatment adherence is a significant factor in clinical outcomes for PCOS patients.

## Supporting information

S1 Fig PRISMA checklist. (DOC)

S1 Table Search strategy (PubMed). (DOCX)
