# Supplementary material for: Adherence to treatment for polycystic ovarian syndrome: A systematic review
Source: PLoS One. 2020 Feb 13;15(2):e0228586. doi: 10.1371/journal.pone.0228586 (PMC7017995; doi:10.1371/journal.pone.0228586)
Supplement: S1 Table — (DOCX) [file pone.0228586.s002.docx]

**S1 Search Strategy (PubMed)**

| #1 | Polycystic ovarian syndrome [mh] |
| --- | --- |
| #2 | Adherence |
| #3 | Patient compliance |
| #4 | #1 and #2 |
| #5 | #1 and #3 |
